# In Silico-Assisted Isolation of *trans*-Resveratrol and *trans*-ε-Viniferin from Grapevine Canes and Their Sustainable Extraction Using Natural Deep Eutectic Solvents (NADES)

**DOI:** 10.3390/foods12224184

**Published:** 2023-11-20

**Authors:** Mats Kiene, Malte Zaremba, Hendrik Fellensiek, Edwin Januschewski, Andreas Juadjur, Gerold Jerz, Peter Winterhalter

**Affiliations:** 1 Institute of Food Chemistry, Technische Universität Braunschweig, Schleinitzstraße 20, 38106 Braunschweig, Germany; m.kiene@tu-braunschweig.de (M.K.); e.januschewski@dil-ev.de (E.J.); g.jerz@tu-braunschweig.de (G.J.); 2 German Institute of Food Technologies, Chemical Analytics, Prof.-von-Klitzing-Straße 7, 49610 Quakenbrück, Germany; a.juadjur@dil-ev.de

**Keywords:** grapevine canes, stilbenoids, resveratrol, viniferin, high-performance countercurrent chromatography, natural deep eutectic solvents, conductor-like screening model for real solvents

## Abstract

Grapevine canes are an important source of bioactive compounds, such as stilbenoids. This study aimed to evaluate an in silico method, based on the Conductor-like Screening Model for Real Solvents (COSMO-RS) to isolate stilbenoids from a grapevine cane extract by *offline heart-cut* high-performance countercurrent chromatography (HPCCC). For the following extraction of resveratrol and ε-viniferin from grapevine canes, natural deep eutectic solvents (NADES) were used as an environmentally friendly alternative to the traditionally used organic solvents. In order to evaluate a variety of combinations of hydrogen bond acceptors (HBAs) and hydrogen bond donors (HBDs) for the targeted extraction of stilbenoids, COSMO-RS was applied. In particular, ultrasonic-assisted extraction using a solvent mixture of choline chloride/1,2-propanediol leads to higher extraction yields of resveratrol and ε-viniferin. COSMO-RS calculations for NADES extraction combined with HPCCC biphasic solvent system calculations are a powerful combination for the sustainable extraction, recovery, and isolation of natural products. This in silico-supported workflow enables the reduction of preliminary experimental tests required for the extraction and isolation of natural compounds.

## 1. Introduction

Wine production from *Vitis vinifera* spp. (Vitaceae) generates tremendous amounts of organic waste, such as skins, pulp, seeds, and wine lees. In addition, during the annual pruning, woody residues, such as the grapevine canes, are obtained. Grapevine canes are a good source for bioactive compounds whose economic potentials have been evaluated by Rayne et al. [1]. They contain large amounts of extractable phytochemicals with antioxidative, cardioprotective, and antiproliferative activities, such as the stilbenoids *trans*-resveratrol and *trans*-ε-viniferin [2,3,4,5]. The application of these bioactives for dietary supplements or cosmetic products has led to a strong scientific and economic interest in novel solvent-free and ecologically safe methods for extraction procedures. In addition to the conventional extraction of resveratrol from grapevine canes with ethanol/water mixtures [6], the use of natural deep eutectic solvents (NADES) for the solvent-free recovery of resveratrol from the roots of *Arachis hypogaea* (Fabaceae) and *Polygonum cuspidatum* (Polygonaceae), as well as *Gnetum gnemon* (Gnetaceae) seeds, has been previously described and the potential of these innovative extraction agents has been clearly revealed [7,8,9]. Most recently, Petit et al., described an ultrasound-assisted method for the extraction of polyphenols, including resveratrol and ε-viniferin, from Pinot noir grapevine canes (*Vitis vinifera*, Vitaceae) with three choline chloride-based NADES systems, and Duarte et al. used three levulinic acid-based DES for the extraction of polyphenols from the vine shoots of 13 different *Vitis* sp. [10,11].

**Figure 1:**
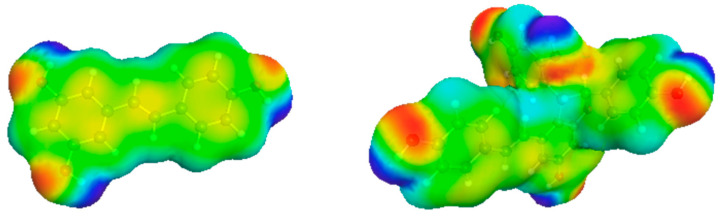
Charge density surface of *trans*-resveratrol (**left**) and *trans*-ε-viniferin (**right**). The different colors describe the charge density distribution on molecular surfaces (blue = electro-positive region, green = neutral region, red = electro-negative region).

NADES are a special category of deep eutectic solvents (DES), which were first described by Abbott et al. [12]. DES are defined as liquid mixtures of pure components whose melting temperatures are below that of an ideally eutectic mixture, and thus must deviate strongly from the ideal state [13,14]. The melting point depression results from strong intermolecular, non-covalent interactions, consisting mainly of hydrogen bonding [15]. These effects lead to the formation of an energetically favored supramolecular lattice in which water can be incorporated up to a certain level [16]. The extension of DES to natural deep eutectic solvents, introduced by Choi et al., (2011), refers to components such as organic acids, amino acids, or carbohydrates, which originate naturally from the primary plant metabolism and are present in large quantities there [17]. NADES are generally formed by mixing hydrogen bond acceptors (HBAs) and hydrogen bond donors (HBDs). It should be noted that the hydrogen-bonding interactions between HBAs and HBDs become weaker with increasing dilution by water [18]. Quaternary ammonium salts and amino acids serve as HBAs, and carbohydrates or organic acids are used as HBDs. Compounds containing carboxyl, alcohol, amino, and keto groups act as both HBAs and HBDs [19]. NADES are prepared by stirring the components weighed in a certain ratio at an elevated temperature or by removing the components dissolved in excess water by lyophilization until a clear liquid is obtained [19,20]. NADES are of scientific interest as they can replace conventional organic solvents as an environmentally friendly alternative. Their attributes are a lack of toxicity, flammability, their biodegradability, stability and low vapor pressure, ease of extraction, manufacturability, and lower costs [21]. In addition, NADES show advantages in terms of improving extraction yields or stabilizing and activating enzymes in biocatalytic reactions [22,23]. Another reason for the increasing scientific interest is their character as designer solvents. Through the choice of the starting compounds, the molar HBA/HBD ratio, and water content, properties of NADES are adjustable, including, most importantly, their polarity and viscosity. Thus, depending on the application, NADES can be tailored to the target compounds to be extracted and the sample material, leading to a multitude of possible, applicable NADES systems [24]. In order to select promising NADES mixtures from the multitude of probable combinations of HBAs and HBDs, the in silico calculation model—Conductor-like Screening Model for Real Solvents (COSMO-RS) developed by Klamt and co-workers—can be used for predictions [25,26,27]. Therefore, the charge density distribution on the surface of the NADES mixtures, as well as of the stilbenoids (resveratrol and ε-viniferin, cf. Figure 1) are calculated quantum-chemically with COSMO-RS. Afterwards, the chemical potentials of all pure compounds and the chemical potentials in the liquid phase are calculated. From this, the activity coefficients of the stilbenoids in the NADES mixtures can be calculated, whereby a smaller activity coefficient represents the better solubility of stilbenoids in these NADES. Previously, COSMO-RS was already used to select appropriate NADES for the extraction of catechin from Graševina grape pomace [28], phenolic compounds from *Rosmarinus officinalis* [29], or hydroxytyrosol from *Olea europaea* [30].

For the determination of the extraction yields of the two stilbenoids in NADES extracts from grapevine canes, authentic references were used which were isolated by all-liquid chromatography methods. In earlier studies, ε-viniferin and resveratrol were isolated from *Vitis vinifera* stems by a two-step centrifugal partition chromatography method (CPC) [31,32], or low-speed rotary countercurrent chromatography [3]. In our study, high-performance countercurrent chromatography (HPCCC) was selected as a semi-preparative liquid separation technique, where the adsorption of target compounds can be avoided due to the absence of a solid stationary phase, and where process scale-up is easily possible due to predictable parameter settings [33,34]. The separation principle of liquid–liquid chromatography is based on the differences in the specific partition ratio values (*K_D_*) of compounds between two immiscible phase layers of a solvent system used as a mobile and stationary phase [34]. HPCCC as a hydrodynamic all-liquid system implements a planetary motion of two self-balancing rotating coils equipped with wounded multi-layer Teflon tubing (PTFE tube) as the channels for the chromatographic phases and the separation process. Specifically, HPCCC allows high mobile flow rates and rotation speeds (up to 1600 rpm), resulting in relatively short separation times and good chromatographic peak resolution [35,36,37]. For obtaining a suitable solvent system for the separation of the two stilbenoids, COSMO-RS was used to calculate the so-called liquid–liquid equilibrium (LLE) and solute partition ratios (*K_D_*) for ε-viniferin and resveratrol. COSMO-RS calculation only requires the molecular structure of the solute molecules and the specific solvent system’s compositional values in the two-phase layers. The intermolecular forces of solvents in a liquid system can be described as pairwise interactions of surface segments representing the charge density distribution on the molecular surface calculated by COSMO-RS. In the software tool, this leads to a highly efficient and rather fast calculation routine of the chemical potentials of any compound in a two-phase solvent system [25,26,27]. Minceva et al., previously demonstrated the ability of COSMO-RS to pre-calculate compound-specific *K_D_* values for HPCCC or FCPC experiments [38,39,40]. Applications for COSMO-RS as a solvent selection tool in all-liquid chromatography by calculating both LLE and *K_D_* values have been described, for example, for the separation of tocopherols with CPC using deep eutectic solvent-based biphasic systems [41], as well as for the separation of flavonoids from *Arachis hypogaea* hulls [42].

In the present study, COSMO-RS calculations are used for the first time to reduce the time-consuming preliminary experiments in the isolation and extraction of resveratrol and ε-viniferin from the side-streams of wine production. One aim of this study is the evaluation of COSMO-RS as a solvent selection tool for the targeted isolation of resveratrol and ε-viniferin from the commercial grapevine-shoot extract Vineatrol^®^30 by HPCCC. In addition, suitable combinations of HBAs and HBDs will be designed by COSMO-RS to develop a green extraction method for the simultaneous extraction of resveratrol and ε-viniferin from grapevine canes. Finally, we compare how well the solubility trends calculated by COSMO-RS fit with the measured extraction rates.

## 2. Materials and Methods

### 2.1. Chemicals

Double-deionized water (Nanopure^®^, Werner GmbH, Leverkusen, Germany) was used. Ethanol (HPLC grade), methanol (HPLC grade), *n*-hexane (HPLC grade), and acetic acid (LC-MS grade) were purchased from Fisher Scientific (Loughborough, UK). Acetonitrile (HPLC and LC-MS grade) were obtained from Honeywell Specialty Chemicals (Seelze, Germany). Formic acid (HPLC grade) was purchased from VWR Int. S.A.S (Darmstadt, Germany). Ethyl acetate (analytical grade) was purchased from Carl Roth (Karlsruhe, Germany). For TLC, spray reagent anisaldehyde (>98%) from Merck (Darmstadt, Germany) and chloroform (HPLC grade, VWR Int. S.A.S, Darmstadt, Germany) were used. Acetone-*d_6_* (99.9% D) from Deutero GmbH (Kastellaun, Germany) and internal standard tetramethylsilane (TMS) from Sigma-Aldrich (Deisenhofen, Germany) were used for NMR spectroscopic measurements. For NADES preparation, betaine (98%) and urea (99.5%) were purchased from Fisher Scientific (Loughborough, UK). d-Sorbitol (>98%) and d-glucose (99%) were obtained from Carl Roth (Karlsruhe, Germany). 1,4-Butanediol (>98%), choline chloride (>98%), and 1,2-propanediol (99%) were purchased from Sigma-Aldrich (Deisenhofen, Germany). *Trans*-resveratrol standard (>98%) was obtained from Carl Roth (Karlsruhe, Germany) and *trans*-ε-viniferin was isolated from Vineatrol^®^30 (>94%, *λ* 280 nm). All samples prepared for UHPLC and LC-MS analysis were filtered through 0.2 µm PTFE syringe filters from Agilent Technologies (Waldbronn, Germany).

### 2.2. Plant Materials

Grapevine canes (“Sauvignon blanc” collected in Neustadt an der Weinstraße, Germany) were lyophilized until a constant weight was reached (freeze dryer Christ Alpha 2–4, Osterode, Germany). The lyophilized samples were ground by a cutting mill equipped with a parallel section rotor and a bottom sieve with trapezoid holes of 1.5 mm (Retsch SM 1, Haan, Germany). For HPCCC study, the Vineatrol^®^30 grapevine extract from Breko GmbH (Bremen, Germany) was used.

### 2.3. Computational Calculations by COSMO-RS

#### 2.3.1. Calculation of the Phase Equilibrium and Compound-Specific *K_D_* Values for HPCCC Separation B

To verify whether ε-viniferin and resveratrol could be separated using *offline heart-cut* HPCCC (separation B), several solvent systems were pre-evaluated by applying the Conductor-like Screening Model for Real Solvents (COSMO-RS). This model was used to calculate the partition ratio (*K_D_*) values of the two target compounds. In the initial stage, the chemical structures of ε-viniferin and resveratrol were retrieved from ChemSpider [43] as SMILES (simplified molecular input line entry specification) and entered into the software BIOVIA COSMOconfX (version 22.0.0, Dassault Systèmes, Vélizy-Villacoublay, France). The conformers of ε-viniferin and resveratrol were calculated in COSMOconfX using the Becke-Perdew functional (BP), a triple-zeta valence polarization with diffuse functions (TZVPD) and a fine-grid marching tetrahedron cavity (FINE) template. The template includes full geometry optimization with the density functional theory (DFT) at the BP-TZVP level, with a consecutive BP-def2-TZVPD single-point calculation, and a FINE cavity for the COSMO calculation. The conformers were considered as a Boltzmann-weighted mixture of conformers for the calculations. The maximum number of conformers from the geocheck clustering was set at 40, and 75 was the maximum number of conformers. Calculated structures were verified to be true minima using vibrational frequency analysis. The COSMO files of *n*-hexane, ethyl acetate, methanol, and water have been retrieved from the database (BIOVIA COSMObaseEditor, version 21.0.0, Dassault Systèmes).

The LLE compositions of each phase for solvent systems were calculated by the liquid extraction module for a temperature of 20 °C with the software BIOVIA COSMOthermX (version 22.0.0, Dassault Systèmes) [44] using the BP_TZVPD_FINE_22.ctd parameterization (cf. Supplementary Materials Table S1). In the following step, the calculated LLEs were used to calculate the *K_D_* values of ε-viniferin and resveratrol for a fixed separation temperature at 28 °C using the liquid extraction module of the BIOVIA COSMOthermX software with the BP_TZVP_22.ctd parameterization. All conformers of the compounds were used for both calculation steps.

COSMO-RS enables the computation of the activity coefficient ($\gamma_{i}^{\infty ,s}$) of solute *i* when infinitely diluted in solvent *s*, which is utilized to determine the *K_D_* value (Equation (1)):

(1) $$\gamma_{i}^{\infty ,s}=\exp\;\left(\left(\mu_{i}^{s}\;-\;\mu_{i}\right)/\mathrm{RT}\right)$$

where $\mu_{i}^{s}$ is the chemical potential of the solute *i* in the solvent *s* and $\mu_{i}$ is the chemical potential of pure solute *i*.

The *K_D_* value is defined as the concentration of solute *i* in the stationary upper phase ($c_{i}^{u}$) divided by the concentration of solute *i* in the mobile lower phase ($c_{i}^{l}$) and can also be expressed as a ratio of the activity coefficients of the solute in infinite dilution ($\gamma_{i}^{\infty }$) multiplied by the molar volumes of the phases (*v*) (Equation (2)). The phase volume quotient $\frac{v_{l}}{v_{u}}$ was composed of calculated molar volumes by COSMO-RS [39,45,46,47]:

(2) $$K_{D}=\frac{c_{i}^{u}}{c_{i}^{l}}=\frac{\gamma_{i}^{\infty ,l}}{\gamma_{i}^{\infty ,u}}\times \frac{v_{l}}{v_{u}}$$

The solvent systems tested by COSMO-RS calculations with the ability to separate ε-viniferin and resveratrol are listed in Table 1.

#### 2.3.2. Calculation of Compound-Specific Activity Coefficients for Screening of Suitable Combinations of HBAs and HBDs for Stilbenoid Extraction

In a screening, the suitability of HBA and HBD combinations as potential NADES-based extractants for resveratrol and ε-viniferin was evaluated using COSMO-RS, which allowed for calculation of the activity coefficient (*γ*) of the compounds in different NADES systems (cf. Equation (1) in Section 2.3.1). The software BIOVIA COSMOconf (version 21.0.0) was used to calculate the COSMO files of the compounds resveratrol, ε-viniferin, betaine, and choline chloride, following the workflow presented in Section 2.3.1. The COSMO files of the other compounds have been retrieved from the database (BIOVIA COSMObaseEditor, version 21.0.0). For modeling HBAs choline chloride, the so-called *ion pair* approach was used. Thereby, the COSMO-RS-optimized structure of choline chloride could be described as a single non-dissociated molecule [48]. The organic acids were treated as protonated compounds. NADES were treated as binary mixtures of HBDs and HBAs at different stoichiometric ratios (3/1, 2/1, 1/1, 1/2, and 1/3) within the framework of COSMO-RS. For this approach, only the conformer with the lowest energy level of resveratrol, ε-viniferin, HBAs, as well as HBDs, was used, allowing for screening with a large number of potentially suitable NADES systems in a short time. Therefore, the software BIOVIA COSMOthermX (version 21.0.0) [44] with BP_TZVPD_FINE_21.ctd parameterization was used to calculate the activity coefficient of resveratrol and ε-viniferin in NADES at 25 °C and infinite dilution with 0–70 wt% of water. As the activity coefficient decreases, the tested HBA and HBD combination is postulated to be better able to solubilize and, therefore, to extract resveratrol and ε-viniferin, respectively [29].

### 2.4. HPCCC Apparatus and Separation Procedure

For isolation of ε-viniferin and resveratrol, a two-step isolation protocol employing a high-performance countercurrent chromatograph—HPCCC (model Spectrum, Dynamic Extractions Ltd., Tredegar, UK)—was used. The multilayer coil planet *J*-type HPCCC centrifuge with two self-balanced different-sized columns consisted of two bobbins, each equipped with one semi-preparative multi-layer polytetrafluoroethylene coil (62.5 mL, 1.6 mm tube i.d.). For the two HPCCC experiments, both semi-preparative coils were connected in series (125 mL). Solvents were pumped with a preparative LC pump K-501 (Knauer Gerätebau GmbH, Berlin, Germany) and elution was monitored at *λ* 280 nm with a Well-Chrom Spectro-Photometer K-2501 detector (Knauer Gerätebau GmbH, Berlin, Germany). The temperature was kept constant at 28 °C using a ULK 2002 recirculating chiller from Fryka-Kältetechnik GmbH (Esslingen, Germany). For chromatographic data acquisition, Eurochrom 2000 software (Windows version) from Knauer Gerätebau GmbH (Berlin, Germany) was used.

For separation A, the two-phase solvent system *n*-hexane/ethyl acetate/methanol/water (1.0/2.0/1.0/2.0; *v*/*v*/*v*/*v*) (HEMWat) was used, which was previously described for separation of the Vineatrol^®^30 extract [3,49]. Prior to the chromatographic process, the two-phase solvent system was freshly prepared. The HEMWat mixture was equilibrated in a separatory funnel at ambient temperature. The resulting two-phase layers were separated and shortly degassed by ultra-sonication. The separation was done in the *head-to-tail* reversed phase operation mode (mobile phase: aqueous lower phase; stationary phase: organic upper phase). The HPCCC was filled with the stationary phase, rotation was set to 1600 rpm, and the mobile phase was pumped at a flow rate of 4.0 mL/min. After reaching the hydrodynamic equilibrium (mobile phase break-through), the grapevine extract Vineatrol^®^30 was dissolved in both solvent phases (1/1; *v*/*v*) at a concentration of 120 mg/mL, filtered over a Chromafil Xtra GF-100/25 fiberglass membrane disc filter (1 µm pore size, 25 mm i.d., Macherey and Nagel, Düren, Germany), and injected using a 5.0 mL sample loop (equiv. to 600 mg of dry crude extract). The volume of stationary phase displaced during the equilibration procedure (*V_M_*: mobile phase take-up) was measured and subtracted from the total coil volume (*V_C_*). The stationary phase content (*V_S_*) remaining in the coil column system can be converted to the solvent system retention value (*S_F_* [%] = *V_S_*/*V_C_* × 100). In the case of separation A, *S_F_* was 68%. This value was corrected by the periphery volume *V_ext_* of tubings (cf. Supplementary Materials Section S2).

The co-eluting compounds, ε-viniferin and resveratrol (*R_t_* = 52–65 min, *K_D_* = 2.18), were collected, dried by lyophilization, and transferred to separation B via a manual *offline heart-cut* procedure. For separation B, the solvent system selection was supported by COSMO-RS, as described in Section 2.3.1, which led to the HEMWat system with the more polar composition 1.0/1.5/1.0/1.5 (*v*/*v*/*v*/*v*). HPCCC system filling and sample injection were carried out as described above. The mobile phase was pumped at a flow rate of 3.0 mL/min and ~100 mg of the *heart-cut fraction* was injected by a 5.0 mL sample loop (20 mg/mL). In case of separation B, 76% for *S_F_* was calculated. Fractions were collected using B-type racks of a Pharmacia Superfrac (Uppsala, Sweden) with a frequency of 1 min/fraction and were analyzed by TLC (cf. Section 2.5). Pure ε-viniferin and resveratrol were isolated during HPCCC separation B and directly used for 1D/2D-NMR experiments (cf. Supplementary Materials Section S2).

### 2.5. Analysis of HPCCC Fractions by TLC

The HPCCC fractions were analyzed on normal phase silica gel TLC plates (SiO_2_-60 F_254_, Merck GmbH, Darmstadt, Germany). The plates were developed with the solvent system chloroform/ethyl acetate/methanol/water (25/55/5/1; *v*/*v*/*v*/*v*) [3]. Results were visualized by the universal anisaldehyde spray reagent, consisting of anisaldehyde, concentrated sulfuric acid, and glacial acid [50]. Finally, for compound visualization, the plates were heated (105 °C) in a drying oven.

### 2.6. Preparation of Natural Deep Eutectic Solvents

For NADES preparation, all solids were dried in a drying oven at 70 °C for 12 h and then stored in a desiccator until use. The preparations of the NADES systems were carried out using the heating method, according to Dai et al., (2013) [16]. For this purpose, HBAs and HBDs were weighed in a specific molar ratio and, if necessary, a certain weight percentage (wt%) of water was added. The components were then stirred on a magnetic stirrer with heating function at 70–80 °C until a homogeneous, clear liquid was formed. Abbreviations and compositions of manufactured NADES are given in Table 2.

### 2.7. Ultrasonic-Assisted NADES-Extraction of Resveratrol and ε-Viniferin

For extraction, approximately 500 mg of the ground grapevine canes were placed in centrifuge tubes, mixed with 5 g of NADES or organic extraction solvents, and mixed with a vortexer. Subsequently, the solutions were extracted using an ultrasonic homogenizer equipped with a 1/8″ ultrasonic horn (Branson Ultrasonics Sonifier S450A, Danbury, CT, USA) with an output control of 20% for 4.5 min. The extract was centrifuged at 8000 rpm for 5 min. After completion of centrifugation, the supernatant was removed by fiberglass membrane disc filter (1 µm pore size, 25 mm i.d., Chromafil Xtra GF-100/25, Macherey and Nagel, Düren, Germany), transferred to volumetric flasks, and made up to a defined volume with water/acetonitrile (1/1; *v*/*v*). The extraction was performed in triplicates for the grapevine cane powder. For determination of the extraction content, a calibration curve for *trans*-resveratrol and *trans*-ε-viniferin were prepared. The calibration curves were recorded in seven different concentrations, i.e., 20.0, 10.0, 8.0, 2.5, 1.0, and 0.5 mg/L for resveratrol, and 20.0, 10.0, 5.0, 2.5, 1.0, 0.5, and 0.1 mg/L for ε-viniferin. The extraction content of stilbenoids in grapevine canes was reported as milligrams per gram of dry weight (DW).

### 2.8. Successive Ultrasonic-Assisted Extraction

For determining the extraction yield, a successive extraction based on the procedure of Ewald et al., (2017) was performed [6]. An amount of 2.5 g of the ground grapevine canes was weighed into a centrifuge tube. Then, 20 mL of the extraction solution consisting of ethanol and water (80/20; *v*/*v*) was added and extracted with an ultrasonic homogenizer equipped with a 1/8″ ultrasonic horn (Branson Ultrasonics Sonifier S450A, Danbury, CT, USA) with an output control of 20% for 4.5 min. Each extraction step was followed up by a centrifugation step at 8000 rpm for 5 min (Hettich Universal 30F, Lauenau, Germany). The supernatants were combined and made up to 100 mL. The extraction was repeated a total of four times. The extract was dried under nitrogen and afterwards reconstituted with ethanol/water mixture (80/20; *v*/*v*), resulting in 10-fold concentrated solutions.

### 2.9. UHPLC-UV Analysis

For quantification of stilbenoids, an UHPLC system from Agilent Technologies (Waldbronn, Germany), with a high-speed pump (1290 Infinity II series), multicolumn thermostat (1290 Infinity II series), vial sampler (1290 Infinity II series), and a diode array detector (1290 Infinity II series) was used. Analysis and data collection were made by Open Lab CDS 3.4 (Agilent Technologies, Waldbronn, Germany). Separation was achieved on a Zorbax Eclipse Plus C18 column (1.8 µm, 50 × 2.1 mm, Agilent Technologies, Waldbronn, Germany). The mobile phase consisted of water/acetonitrile/formic acid (100/10/0.1; *v*/*v*/*v*) (A) and acetonitrile (B). The separation was carried out at 60 °C with a flow rate of 0.4 mL/min, under the following conditions: 0 min (0% B), 1.73 min (0% B), 2.73 min (16% B), 6.2 min (45% B), 6.4 min (0% B), and 7.2 min (0% B). *Trans*-resveratrol and *trans*-ε-viniferin were quantified at *λ* 306 and 324 nm, respectively. The quantification parameters of the UHPLC-UV methodology are listed in Table 3.

### 2.10. HPLC-UV-MS Analysis

For qualification and peak identification purposes, an HPLC-ESI-MS system consisting of a binary HPLC pump (1100 series) and autosampler (1200 series) from Agilent Technologies (Waldbronn, Germany) equipped with an LC-ESI-MS/MS ion-trap system (HCT Ultra ETD II, Bruker Daltonics, Bremen, Germany) was used. Mass spectra were recorded in the negative ionization mode with the capillary voltage set at 3500 V, end plate at −500 V, and capillary exit at −115.0 V. Drying gas was nitrogen at 330 °C and 10.0 L/min flow rate with nebulizer pressure of 50 psi, target mass setting *m*/*z* 350, scan range from *m*/*z* 100–2000 in Ultra-Scan mode, and fragmentation amplitude of 1 V. Compass Hystar Software (version V. 3.2, Bruker Daltonics) was used for analysis and data collection. HPLC separation was carried out on a C18-column (Aqua 3u, 100 Å, 3 μm, 150 mm × 2.0 mm i.d.) from Phenomenex (Aschaffenburg, Germany) with a guard column of the same material at a flow rate of 0.20 mL/min. The mobile phase consisted of 2% aqueous acetic acid (*v*/*v*) (A) and acetonitrile (B). HPLC conditions for ESI-MS/MS analysis were 0 min (20% B), 20 min (30% B), 40 min (50% B), 50 min (80% B), 55 min (80% B), 60 min (20% B), and 70 min (20% B).

### 2.11. Spectroscopic Measurements

The isolated compounds were identified by one- and two-dimensional NMR spectroscopic experiments (^1^H, ^13^C, HSQC, HMBC) on a Fourier 300 spectrometer (Bruker Biospin, Ettlingen, Germany) with a probe head at 300.1 (^1^H) and 75.5 (^13^C) MHz, respectively. Samples were recorded in acetone-*d_6_* and referenced to tetramethylsilane (TMS). Chemical shifts *δ* are reported in parts per million [ppm], coupling constants *J* in Hertz [Hz].

### 2.12. Statistical Analysis

Experimental results were expressed as the mean value ± standard deviation (SD) (n = 3). Statistical analyses were performed using the software OriginPro 2022 (OriginLab Corporation, Northampton, MA, USA), version 9.9.0.225. The significance of difference (*p* < 0.05) was assessed using ANOVA followed up by Tukey’s HSD test.

## 3. Results and Discussion

### 3.1. COSMO-RS-Supported Isolation of ε-Viniferin and Resveratrol by Two-Step HPCCC Separation

**Figure 2:**
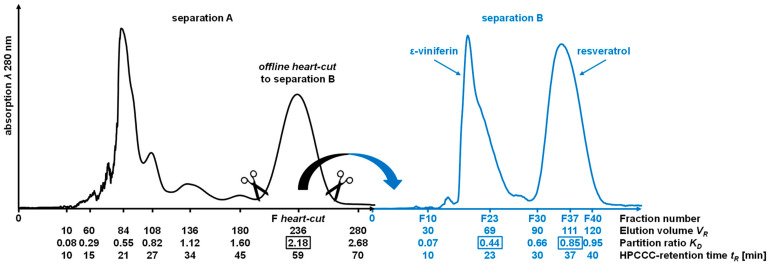
UV chromatogram monitored at *λ* 280 nm of the Vineatrol^®^30 HPCCC separation A (black colored chromatogram) with the HEMWat system 1.0/2.0/1.0/2.0 (*v*/*v*/*v*/*v*) and separation B (HEMWat system 1.0/1.5/1.0/1.5; *v*/*v*/*v*/*v*) after *offline heart-cut* (blue-colored chromatogram).

In order to obtain reference substances for the extraction experiments with natural deep eutectic solvents, the stilbenoids ε-viniferin and resveratrol were isolated by HPCCC. In the first step, the enrichment of the two target components was achieved by initial pre-separation (separation A), as shown in Figure 2. For this pre-separation, the solvent system consisting of *n*-hexane/ethyl acetate/methanol/water (1.0/2.0/1.0/2.0; *v*/*v*/*v*/*v*) was used [49]. In total, 600 mg of Vineatrol^®^30 was separated by HPCCC in *head-to-tail* mode within 70 min. The co-eluting ε-viniferin and resveratrol peak (*R_t_* = 52–65 min, *V_R_* = 236 mL, *K_D_* = 2.18) was collected and transferred after drying to separation B via *offline heart-cut*. The separation factor *α* for the *heart-cut fraction* to previously peak with a *K_D_* value of 1.60 was about 1.37 and resolution *R_S_* was 1.42.

Subsequently, COSMO-RS was used to find a suitable solvent system for HPCCC separation from the *heart-cut fraction* containing ε-viniferin and resveratrol. For this purpose, the phase equilibria for the solvent systems 1–4 (cf. Table 1) were calculated by COSMO-RS using the liquid-extraction module. Afterwards, the compound-specific *K_D_* values were calculated (cf. Table 4).

The *K_D_* values of the target compounds in liquid–liquid countercurrent chromatography should be in the range of 0.4 ≤ *K_D_* ≤ 2.5 [51]. The *K_D_* values for the ε-viniferin and resveratrol of systems 2–4 were in the postulated range. System 4 was not used because its *α* value of 1.07 was too small compared to systems 2 and 3. The calculated *α* value of the pair, ε-viniferin/resveratrol, is smaller in system 3 compared to system 2, but due to the higher calculated *K_D_* value of ε-viniferin (0.57), there should be no co-elution of any other polar components from the *heart-cut fraction*. For this reason, the solvent system consisting of *n*-hexane/ethyl acetate/methanol/water (1.0/1.5/1.0/1.5; *v*/*v*/*v*/*v*) (system 3) was used for separation B.

By using system 3, 100 mg of the *heart-cut fraction* were separated by HPCCC in *head-to-tail* mode, yielding a total of 60 fractions in *elution* mode. The HPCCC fractions were screened by TLC and fractions were pooled based on the different compound spots (cf. Supplementary Materials Figure S2). As shown in Figure 2, the dimer ε-viniferin (fractions 18–72, *V_R_* = 69 mL, *K_D_* = 0.44) eluted at smaller retention volumes (*V_R_*) compared to resveratrol (fractions 33–40, *V_R_* = 111 mL, *K_D_* = 0.85) in the HPCCC separation B. The *α* value of the pair is 1.94 and baseline separation was achieved (*R_S_* = 1.75). The experimental *K_D_* value [52] of ε-viniferin differed to those from the COSMO-RS calculation (∆ = 0.13), but for resveratrol, the calculated *K_D_* value was equal to the experimental value. At this stage, pure ε-viniferin (19 mg) and resveratrol (20 mg) with purities of 94% and 99%, respectively, were obtained using this two-step HPCCC separation method (UHPLC-UV analysis *λ* 280 nm). The LC-ESI-MS and NMR spectral data of the two isolated compounds are given in the Supplementary Materials Section S2. This COSMO-RS-supported two-step HPCCC method is characterized by the fact that ε-viniferin and resveratrol were obtained at high purities. The isolated amounts of ε-viniferin (3.2%) and resveratrol (3.3%) offer good potentials for scale-up using all-liquid CCC devices with larger coil column volumes. It was also shown that the COSMO-RS calculation can be used to support the isolation of natural compounds by means of HPCCC.

### 3.2. COSMO-RS-Supported Selection of HBAs and HBDs for Stilbenoid Extraction

Different combinations of HBAs and HBDs have a tremendous influence on the physicochemical properties of the NADES. These properties, such as polarity or solution capability, will influence their extraction efficiency.

**Figure 3:**
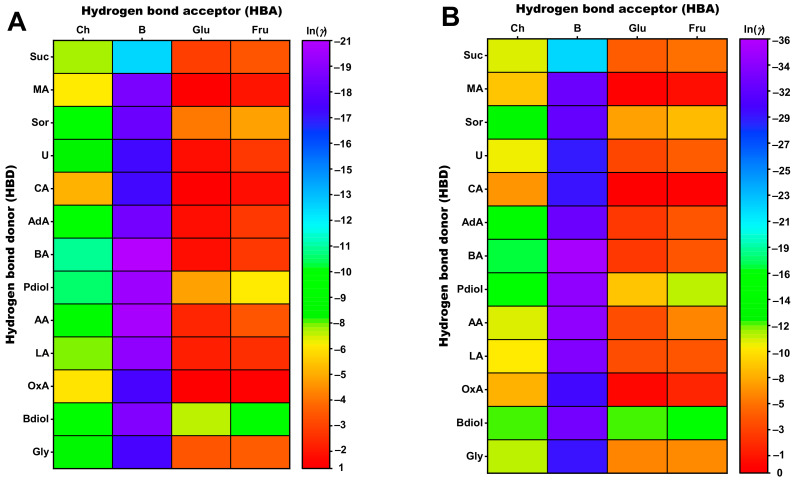
Heat map of the activity coefficients ln *γ* calculated using COSMO-RS (25 °C) of (**A**) resveratrol and (**B**) ε-viniferin. The molar ratio for choline chloride and betaine-based systems is 3/1, while that for glucose- and fructose-based systems is 1/3. The water content for all NADES is 0 wt%. Abbreviations: AA, acetic acid; AdA, adipic acid; BA, benzoic acid; B, betaine; Bdiol, 1,4-butanediol; Ch, choline chloride; CA, citric acid; Fru, fructose; Glu, glucose; Gly, glycerol; LA, lactic acid; MA, malic acid; OxA, oxalic acid; Pdiol, 1,2-propanediol; Sor, sorbitol; Suc, sucrose; U, urea.

In this experimental approach, 52 NADES with four different HBAs (choline chloride, betaine, glucose, and fructose) were selected in advance by the in silico method COSMO-RS based on their ability to extract the stilbenoids resveratrol and ε-viniferin. The suitability of HBA and HBD combinations as potential NADES extractants for resveratrol and ε-viniferin was calculated using the COSMO-RS implemented activity coefficient *γ*. COSMOtherm reports the activity coefficient as ln *γ*, and as this value becomes smaller, the tested combination of HBAs and HBDs is better able to solubilize and extract resveratrol and ε-viniferin, respectively [29]. The calculations have been carried out with different molar ratios and water contents for each combination of HBAs and HBDs. The heat map in Figure 3 displays a portion of around 1200 different combinations. In this model for COSMOtherm, HBAs and HBDs enter the calculation as solids for simplification, assuming that the mixture of both substances, at a defined molar ratio and a certain water content, forms a supramolecular liquid dissolving the target substances. Based on these calculations, the COSMOtherm heat map (Figure 3) gives an overview for the solubility trend of resveratrol (A) and ε-viniferin (B) in the respective NADES systems.

The heat map shows that NADES with betaine as the HBA gave higher solubilities for the two stilbenoids than systems with choline chloride. In this respect, benzoic acid (ln *γ* = −20.5), acetic acid (ln *γ* = −19.9), 1,2-propanediol (ln *γ* = −19.9), and lactic acid (ln *γ* = −19.3) prove to be promising HBDs to prepare a suitable NADES with betaine as the HBA, as they are able to solubilize, e.g., resveratrol. If choline chloride is used as the HBA in these systems, then the activity coefficients of resveratrol for the respective donors increase (HBDs: benzoic acid ln *γ* = −11.0, acetic acid ln *γ* = −8.8, 1,2-propanediol ln *γ* = −10.6, and lactic acid ln *γ* = −7.4). The use of glucose and fructose as the HBAs does not lead to good solubility results of stilbenoids in any of the combinations shown. According to the COSMO-RS calculation, combinations with the HBDs 1,4-butanediol (ln *γ* = −6.9) or 1,2-propanediol (ln *γ* = −4.9) were most suitable for the extraction of resveratrol when glucose is used as the HBA. The HBDs 1,4-butanediol (ln *γ* = −9.1) and 1,2-propanediol (ln *γ* = −6.4) would be the most useful systems when fructose is used as the HBA. When calculating the activity coefficients of ε-viniferin in the NADES systems, the same trend as for resveratrol is observed. These effects could be explained by the calculated sigma profiles of resveratrol and ε-viniferin (Supplementary Materials Figure S1). The sigma profiles of the two stilbenoids were not significantly different, but ε-viniferin has an overall larger COSMO surface area than resveratrol. Both stilbenoids have a large surface area in the apolar region between ±0.01 e/Å^2^, indicating a hydrophobic character, and the maxima in the polar region at −0.017 e/Å^2^ and 0.012 e/Å^2^ indicate the property to induce hydrogen bonding. The calculated activity coefficients of both stilbenoids in the different extractants are listed in Supplementary Materials Tables S2 and S3.

During a trial-and-error preparation of the NADES systems, it was found that some combinations of HBAs and HBDs, such as betaine/benzoic acid or betaine/acetic acid, could not be prepared as the substances recrystallized upon cooling. Moreover, the NADES betaine/1,4-butanediol could only be prepared by adding 30 wt% H_2_O. These observations were in line with previously reported studies showing that, for betaine-based NADES, even a small amount of water is sufficient to form a NADES system. Frequently used NADES with betaine as the HBA have a water content between 30 and 50 wt% [9,28].

For this reason, the NADES used in Section 3.5 represent a compromise between the best possible solubility of the natural products calculated with COSMO-RS and their producibility. A promising system for the extraction of stilbenoids was, therefore, the Ch/Pdiol 1/5 system, which could be prepared without water (ln*_resveratrol_* = −8.4, ln_ε-*viniferin*_ = −14.9). In addition, the selected components were characterized by their wide availability and low cost, as well as their renewability and biodegradability. Further studies on the manufacturing of different NADES systems should be carried out to replace the experimental trial and error essays with in silico methods, if necessary [53,54].

### 3.3. Choosing a Promising Extraction Method

Various extraction methods, e.g., maceration, an ultrasonic bath, and an ultrasonic homogenizer, have been used for the extraction of natural substances from food by-products, such as grapevines [6,55,56]. In previous research, resveratrol and ε-viniferin were extracted with NADES using an ultrasound bath lasting 17–20 min [9,10,11]. A preliminary test was performed to investigate which of the three processes out of maceration, an ultrasonic bath, and an ultrasonic homogenizer is suitable for the extraction of grapevine canes using NADES.

The NADES system used was Ch/Pdiol 1/2, 20 wt% H_2_O. A water content of 20 wt% was chosen for this system to reduce the viscosity, which results from the extensive hydrogen-bonding network between the HBA and HBD [57].

**Figure 4:**
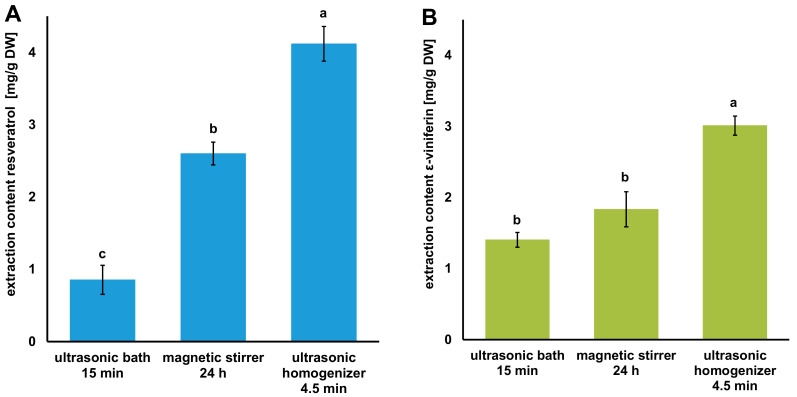
Effect of different extraction methods on the extraction content of (**A**) resveratrol and (**B**) ε-viniferin using the NADES system Ch/Pdiol 1/2, 20 wt% H_2_O (data are expressed as the mean value ± SD; means in the group with different letters (a–c) differ significantly at *p* < 0.05, as measured by Tukey’s HSD Test).

This approach should allow a better comparison of the extraction methods. The contents of resveratrol and ε-viniferin in the NADES extract, after using the three extraction methods, maceration, an ultrasonic bath, and an ultrasonic homogenizer, can be seen in Figure 4. The highest extraction yields of the two stilbenoids in the NADES extract were obtained in 4.5 min using the ultrasonic homogenizer (4.12 ± 0.24 mg/g DW for resveratrol and 3.01 ± 0.13 mg/g DW for ε-viniferin). The yields of the other two extractions with the ultrasonic bath and with stirring were significantly lower (*p* < 0.05). For resveratrol, only 2.60 ± 0.16 mg/g DW was obtained after extraction in the ultrasonic bath, and the lowest yield for extraction under stirring was 0.85 ± 0.20 mg/g DW. Something similar was observed for the different extraction methods of ε-viniferin.

Therefore, to increase the extraction efficiency and ideally minimize the extraction time, ultrasonic probe extraction was performed, which enhances the mass transfer of extractables and increases the extraction rate and efficiency of NADES. Likewise, the viscosity is lowered by the energy input of ultrasonic extraction, which also increases the mass transfer. This is expected to shorten the extraction time, reduce the consumption of NADES, and increase the extraction yield simultaneously [58].

### 3.4. Comparison of Extraction Selectivity of NADES Extract vs. Ethanol/Water Extract by HPLC-ESI-MS/MS Measurements

**Figure 5:**
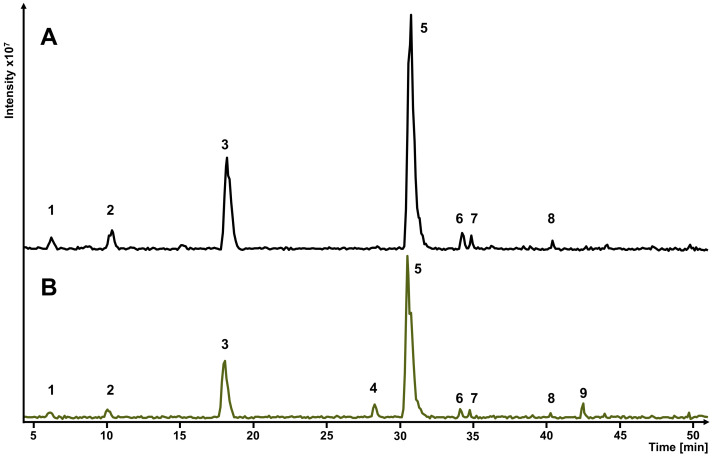
HPLC-ESI-MS base peak chromatogram (neg. mode, BPC 100-2000) of two grapevine cane extracts (**A**) ethanol/water (80/20; *v*/*v*) and (**B**) Ch/Pdiol 1/2, 20 wt% H_2_O (both extracts were obtained by ultrasonic-assisted extraction).

The ultrasonic homogenizer was selected to assist the extraction of grapevine canes with different NADES. The extraction selectivity of the NADES Ch/Pdiol 1/2, 20 wt% H_2_O should first be tested in comparison to an ethanol/water mixture (80/20; *v*/*v*) as the extraction agent. The HPLC-ESI-MS base peak chromatograms (negative mode, BPC 100-2000) of both extracts are shown in Figure 5.

Six stilbenoids could be qualified in both extracts by ESI-MS/MS (Table 5). *Trans*-resveratrol and *trans*-ε-viniferin were found to be the predominant stilbenoids in addition to minor amounts of restrytisol, ampelopsin A, as well as two resveratrol-dimers. Both extraction agents showed a similar selectivity, whereby two more compounds were extracted by using the NADES. These two compounds were a resveratrol-dimer (*m*/*z* 453) and an unidentified compound with the pseudo-molecular ion *m*/*z* 431. Under these aspects, the NADES Ch/Pdiol 1/2, 20 wt% H_2_O fulfilled the requirement to substitute the ethanol/water mixture as the extractant of the grapevine canes, since the obtained composition of the extract is comparable. Finally, we aimed to clarify whether the extraction contents of resveratrol and ε-viniferin obtained after extraction with the different NADES systems were comparable to those extracted with an ethanol/water mixture previously used for the extraction of stilbenoids from grapevine canes.

### 3.5. Extraction of Grapevine Canes with Selected NADES Based on In Silico Calculations by COSMO-RS

**Figure 6:**
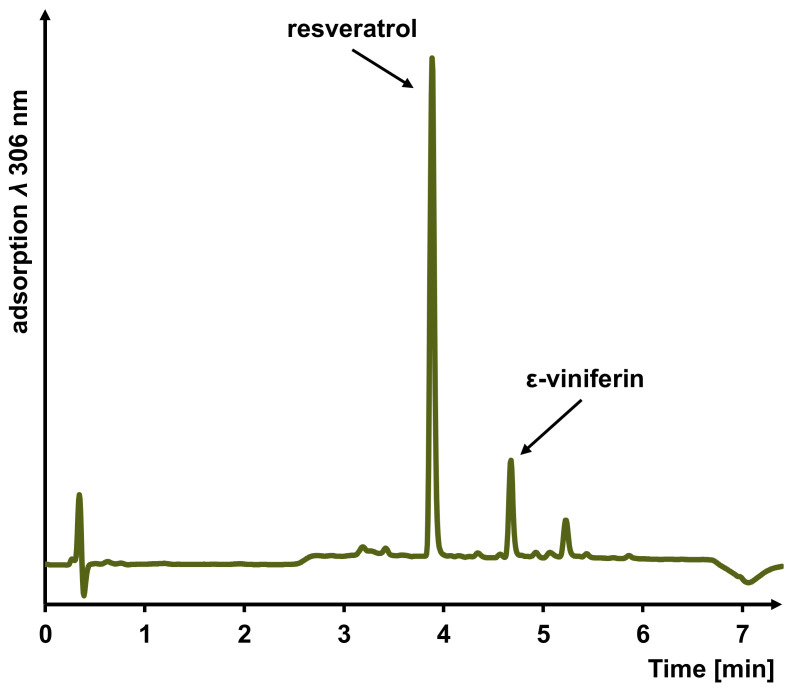
UHPLC-UV chromatogram of a NADES grapevine cane extract (Ch/Pdiol 1/5, 0 wt% H_2_O) monitored at *λ* 306 nm.

Based on the results discussed in Section 3.3, a fast ultrasonic-assisted NADES extraction of the grapevine canes was performed. Additionally, COSMO-RS was used to model appropriate NADES from the large number of possible combinations of HBAs and HBDs that could be used to extract the stilbenoids. The extraction contents of the stilbenoids, resveratrol and ε-viniferin, predominant in the different NADES extracts from grapevine canes, as well as their contents in the ethanol/water extract, were quantified within 7.2 min by a rapid UHPLC-UV method, characterized by its low solvent consumption (Figure 6).

**Figure 7:**
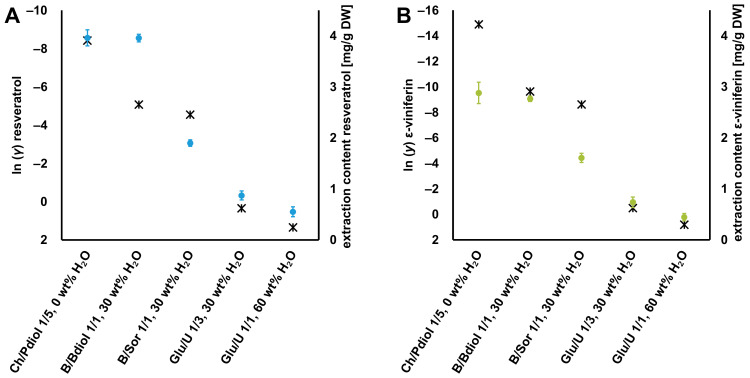
Comparison of COSMO-RS-calculated ln *γ* values (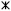) and measured extractions contents (●) of (**A**) resveratrol and (**B**) ε-viniferin. Abbreviations: B, betaine; Bdiol, 1,4-butanediol; Ch, choline chloride; Glu, glucose; Pdiol, 1,2-propanediol; Sor, sorbitol; U, urea.

For these extraction experiments, NADES systems with different activity coefficients were selected to compare the obtained extraction contents of the two stilbenoids with the activity coefficients ln *γ* calculated by means of COSMO-RS. The lower the activity coefficient of a compound calculated with COSMO-RS, the more soluble it is in the respective NADES. In this way, the activity coefficient was used to determine a trend in advance, which gives an indication of the different suitability of the NADES systems for extracting resveratrol and ε-viniferin. Figure 7 shows the ln *γ* as well as the extraction contents of resveratrol and ε-viniferin in different NADES. For this comparison, one NADES without water (Ch/Pdiol 1/5) and three NADES with a 30 wt% water content each were selected (B/Bdiol 1/1, B/Sor 1/1, and Glu/U 1/1). Furthermore, with Glu/U (1/1, 60 wt% H_2_O), a system was included in which the NADES structure should not be formed [18]. This comparison also includes three different HBAs and four different HBDs, so that it was also possible to check how well the solubility trends of the stilbenoids in NADES with different HBAs and HBDs could be calculated by COSMO-RS.

**Figure 8:**
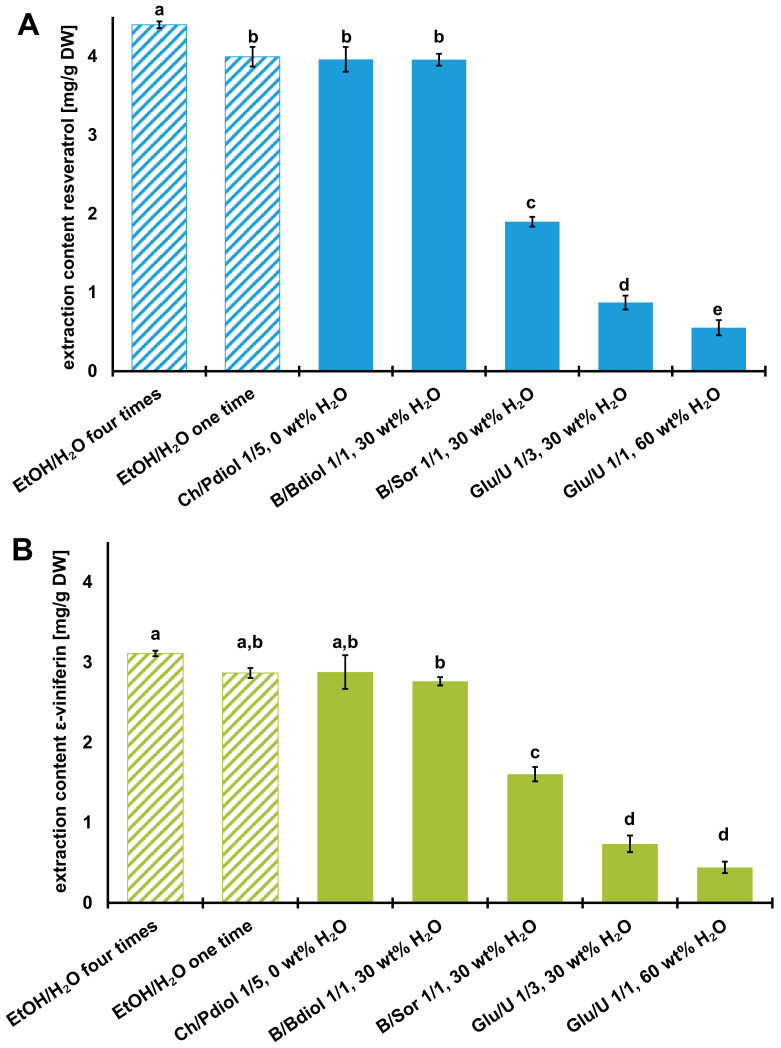
Extraction content of (**A**) resveratrol and (**B**) *ε*-viniferin after extraction of grapevine canes with ethanol/water (shaded) and various NADES systems (data are expressed as the mean value ± SD; means in the group with different letters (a–e) differ significantly at *p* < 0.05, as measured by Tukey’s HSD Test). Abbreviations: B, betaine; Bdiol, 1,4-butanediol; Ch, choline chloride; Glu, glucose; Pdiol, 1,2-propanediol; Sor, sorbitol; U, urea.

The comparison of calculated ln *γ* values by COSMO-RS and the experimental extraction contents of resveratrol and ε-viniferin in different NADES determined by UHPLC-UV was statistically evaluated by Pearson’s coefficient *r* (*α* = 0.05). Due to the negative correlation between ln *γ* and the experimental extraction contents of both stilbenoids, an *r* value < −0.5 indicates a high level of correlation, where 0 indicates the absence of a relationship. The ln *γ* values calculated with COSMO-RS, as well as the experimentally determined values, show the following decreasing trend of the extractive suitability of the NADES systems: Ch/Pdiol 1/5 > B/Bdiol 1/1 > B/Sor 1/1 > Glu/U 1/3 > Glu/U 1/1. The correlation coefficient for resveratrol is −0.91 and for ε-viniferin −0.95, so that the COSMO-RS model could be used to reliably calculate the extractability of the various NADES. Furthermore, it could be confirmed that this method is able to select suitable NADES from a variety of combinations of HBAs and HBDs to extract resveratrol and ε-viniferin from grapevines canes. The extraction contents for resveratrol ranged from 0.55–3.96 mg/g DW and for ε-viniferin from 0.44–2.88 mg/g DW (complete experimental data are shown in Supplementary Materials Table S9). The most suitable NADES for the extraction of resveratrol were the systems with the polyalcohol as HBD Ch/Pdiol 1/5, 0 wt% H_2_O (3.96 ± 0.16 mg/g DW), and B/Bdiol 1/1, 30 wt% H_2_O (3.96 ± 0.08 mg/g DW), which differed significantly from the other systems in their extraction yields, as shown in Figure 8. The NADES system consisting of Glu/U 1/1, 60 wt% H_2_O has the lowest extraction content for resveratrol (0.55 ± 0.10 mg/g DW). These results were consistent with the experimental work of Chen et al., and Petit et al., who also reported that NADES with polyalcohols as HBDs had the highest extraction contents of resveratrol [7,10].

The dimeric stilbenoid ε-viniferin was extracted significantly better with the Ch/Pdiol 1/5, 0 wt% H_2_O system 2.88 ± 0.21 mg/g DW, respectively. It was shown that the ability of the NADES to extract resveratrol and ε-viniferin decreases with increasing water content in the systems. In the NADES Glu/U (1/1, 60 wt% H_2_O), the hydrogen-bonding interactions between the HBA and HBD become weaker, so a solvent with an aqueous character was present that could poorly extract the stilbenoids [18]. The anhydrous NADES Ch/Pdiol 1/5 (e.g., ln *γ_res_* = −8.42) extracted both resveratrol and ε-viniferin the best. Despite the water content of 30%, the NADES B/Bdiol 1/1 (e.g., ln *γ_res_* = −5.08) showed similar extraction properties as Ch/Pdiol 1/5, although the COMSO-RS-calculated ln *γ* value was lower. This could be explained by the fact that the addition of water slightly decreases the viscosity of the NADES, thus increasing mass transfer, which was also observed by Wojeicchowski and co-workers in the extraction of phenolic compounds from rosemary [59].

By comparing the NADES extraction with the conventional ethanol/water extraction (cf. Figure 8), it could be shown that the measured extraction contents for both resveratrol and ε-viniferin did not differ significantly (*p* < 0.05). In further comparison to the four times of extraction of the grapevine canes with the ethanol/water mixture (80/20; *v*/*v*), the single NADES extraction with Ch/Pdiol 1/5, 0 wt% H_2_O, already showed its full potential as an extractant, as 91% of both stilbenoids could be extracted with this system without the optimizing of the extraction procedure.

In the current work, a promising COSMO-RS-supported method for the simultaneous ultrasonic-assisted NADES extraction of resveratrol and ε-viniferin from grapevine canes was developed. In addition, COSMO-RS calculations have been shown to be a powerful tool for modeling appropriate systems from the large number of possible combinations of HBAs and HBDs. The trends of the solubility of the stilbenoids in these systems and the resulting extraction capacity are reliably calculated with COSMO-RS. In subsequent work, it would be of scientific interest to optimize the extraction parameters. Furthermore, there is still a need to develop methods to remove NADES from the extracts, as a comprehensive toxicological and regulatory evaluation of NADES extracts in food and cosmetics has not yet been adequately performed. Increasing scientific interest in NADES extraction processes is related to their direct-to-use application and their specified abilities for target extractions. Through the COSMO-RS-supported selection of the starting compounds, such as the molar HBA/HBD ratio or the water content, the properties of NADES, especially the polarity and viscosity, can be adjusted. Thus, NADES can be tailored to the target compounds to be extracted and the sample material, depending on the application.

## 4. Conclusions

This study has shown that COSMO-RS is a very suitable tool to replace the required preliminary tests needed in the isolation of stilbenoids from grapevine canes by HPCCC and their extraction with NADES. Based on an in silico-supported method for two-step lab-scale isolation, the stilbenoids resveratrol and ε-viniferin were isolated from grapevine-shoot extract in high purities by HPCCC. The scalability of the CCC technique allows it to be used on a larger scale in the future. By using choline chloride/1,2-propanediol 1/5, 0 wt% H_2_O, a water-free NADES system was found, through the COSMO-RS calculations, to be a sustainable and renewable extractant for grapevine canes, with high extraction rates for resveratrol (3.96 ± 0.16 mg/g DW) and ε-viniferin (2.88 ± 0.21 mg/g DW). No significant differences in the extraction yields of the two stilbenoids were observed between the use of choline chloride/1,2-propanediol 1/5, 0 wt% H_2_O and the ethanol/water mixture (80/20; *v*/*v*) as the extractants. These results could also be applied to the extraction of other natural compounds from other side-streams of the food industry with NADES, so that the sustainability of production increases due to a more complete utilization of the products.

## Figures and Tables

**Table 1:** Different *n*-hexane/ethyl acetate/methanol/water (HEMWat) compositions (*v*/*v*/*v*/*v*) used for the COSMO-RS calculations.

Solvent Systems	*n*-Hexane	Ethyl Acetate	Methanol	Water
1	1.00	1.50	1.25	1.25
2	1.00	2.33	1.67	1.67
3	1.00	1.50	1.00	1.50
4	1.00	2.33	1.33	2.00

**Table 2:** Natural deep eutectic solvents and proportion used in this study.

Abbreviation	Component 1 (HBA)	Component 2 (HBD)	Molar Ratio	Water Content (wt%)
Ch/Pdiol	Choline chloride	1,2-Propanediol	1/5	0
B/Bdiol	Betaine	1,4-Butanediol	1/1	30
B/Sor	Betaine	Sorbitol	1/1	30
Glu/U	Glucose	Urea	1/3	30
Glu/U	Glucose	Urea	1/1	60

**Table 3:** Quantification parameters of the UHPLC-UV methodology.

	*trans*-Resveratrol	*trans*-ε-Viniferin
Working range (mg/L)	2.5–20	2.5–20
R^2^	0.9996	0.9993
R_t_ (min)	3.88	4.68
Limit of detection (mg/L)	0.43	0.61
Limit of quantification (mg/L)	1.50	2.10

**Table 4:** COSMO-RS-calculated *K_D_* values and separation factors [*α*] compared with experimental *K_D_* values and HPCCC parameters of separation B (separation factor [*α*] and resolution [*R*].

Solvent Systems Calculated by COSMO-RS	*K_vin_*	*K_res_*	*α_vin/res_*	*R_vin/res_*
1	0.06	0.21	3.50	-
2	0.41	0.65	1.59	-
3	0.57	0.85	1.49	-
4	1.78	1.66	1.07	-
experimental values	0.44	0.85	1.94	1.75

vin: ε-viniferin, res: resveratrol.

**Table 5:** Representative HPLC-ESI-MS/MS profile of grapevine cane NADES extract obtained by ultrasonic-assisted extraction (Ch/Pdiol 1/2, 20 wt% H_2_O).

Nr.	Compound	*t_R_* [min]	Pseudo-Molecular Ion [M-H]^−^ *m*/*z*	Fragment Ions *m*/*z*
1	Restrytisol	6.2	471	349, 255
2	Ampelopsin A	10.1	469	451, 363
3	*trans*-Resveratrol	18.1	227	185, 175, 159
4	Resveratrol-Dimer	28.3	453	435, 411, 359, 347, 289, 253, 225
5	ε-Viniferin	30.5	453	435, 411, 359, 347, 289, 253, 225
6	Resveratrol-Dimer	34.1	453	439, 383, 269
7	unknown	34.7	329	311, 293, 229, 211, 183, 171
8	unknown	40.2	565	387, 372, 357, 289,177
9	unknown	42.4	431	411, 365, 309

## Data Availability

The data presented in this study are available in Supplementary Material.

## Supplementary Materials

The following supporting information can be downloaded at: https://www.mdpi.com/article/10.3390/foods12224184/s1, Section S1: Calculated data by COSMOtherm, Figure S1. Sigma profiles obtained by COSMOthermX of resveratrol and ε-viniferin, Table S1. COSMOthermX phase equilibrium data for HPCCC solvent system selection at 20 °C with TZVPD-FINE parametrization, Table S2. Activity coefficients ln *γ* calculated using COSMO-RS (25 °C) of resveratrol in different NADES, Table S3. Activity coefficients ln *γ* calculated using COSMO-RS (25 °C) of ε-viniferin in different NADES; Section S2: Calculation of countercurrent chromatographic separation parameters, TLC screening of HPCCC fractions, ESI-MS/MS, and NMR data of isolated compounds, Table S4. Compound-specific *K_D_* value from *heart-cut fraction* of HPCCC separation A, Table S5. Target compound-specific *K_D_* values from HPCCC separation B, Figure S2. TLC analysis of the HPCCC fractions, Table S6. ESI-MS/MS data of isolated compounds, Figure S3. Structure of *trans*-resveratrol, Table S7. NMR data of *trans*-resveratrol, Figure S4. ^1^H-NMR spectrum of *trans*-resveratrol, Figure S5. ^13^C-NMR spectrum of *trans*-resveratrol, Figure S6. HSQC spectrum of *trans*-resveratrol, Figure S7. HMBC spectrum of *trans*-resveratrol, Figure S8. Structure of *trans*-ε-viniferin, Table S8. NMR data of *trans*-ε-viniferin, Figure S9. ^1^H-NMR spectrum of *trans*-ε-viniferin, Figure S10. ^13^C-NMR spectrum of *trans*-ε-viniferin, Figure S11. HSQC spectrum of *trans*-ε-viniferin, Figure S12. HMBC spectrum of *trans*-ε-viniferin; Section S3: Experimental data of NADES extraction, Table S9. Extraction contents of resveratrol and ε-viniferin in different extractants. References [35,50,52] are cited in the Supplementary Materials.

## References

[1] Rayne S., Karacabey E., Mazza G. (2008). Grape Cane Waste as a Source of Trans-Resveratrol and Trans-Viniferin: High-Value Phytochemicals with Medicinal and Anti-Phytopathogenic Applications. Ind. Crop. Prod..

[2] Müller C., Ullmann K., Wilkens A., Winterhalter P., Toyokuni S., Steinberg P. (2009). Potent Antioxidative Activity of Vineatrol^®^30 Grapevine-Shoot Extract. Biosci. Biotechnol. Biochem..

[3] Macke S., Jerz G., Empl M.T., Steinberg P., Winterhalter P. (2012). Activity-Guided Isolation of Resveratrol Oligomers from a Grapevine-Shoot Extract Using Countercurrent Chromatography. J. Agric. Food Chem..

[4] Baur J.A., Sinclair D.A. (2006). Therapeutic Potential of Resveratrol: The in Vivo Evidence. Nat. Rev. Drug Discov..

[5] Willenberg I., Michael M., Wonik J., Bartel L.C., Empl M.T., Schebb N.H. (2015). Investigation of the Absorption of Resveratrol Oligomers in the Caco-2 Cellular Model of Intestinal Absorption. Food Chem..

[6] Ewald P., Delker U., Winterhalter P. (2017). Quantification of Stilbenoids in Grapevine Canes and Grape Cluster Stems with a Focus on Long-Term Storage Effects on Stilbenoid Concentration in Grapevine Canes. Food Res. Int..

[7] Chen J., Jiang X., Yang G., Bi Y., Liu W. (2018). Green and Efficient Extraction of Resveratrol from Peanut Roots Using Deep Eutectic Solvents. J. Chem..

[8] Wang J.-D., Fu L.-N., Wang L.-T., Cai Z.-H., Wang Y.-Q., Yang Q., Fu Y.-J. (2021). Simultaneous Transformation and Extraction of Resveratrol from *Polygonum cuspidatum* Using Acidic Natural Deep Eutectic Solvent. Ind. Crop. Prod..

[9] Aryati W.D., Azka K.M., Mun’im A. (2020). Ultrasonic-Assisted Extraction using a Betaine-based Natural Deep Eutectic Solvent for Resveratrol Extraction from Melinjo (*Gnetum gnemon*) Seeds. Int. J. Appl. Pharm..

[10] Petit E., Rouger C., Griffault E., Ferrer A., Renouf E., Cluzet S. (2023). Optimization of Polyphenols Extraction from Grapevine Canes Using Natural Deep Eutectic Solvents. Biomass Convers. Biorefin..

[11] Duarte H., Aliaño-González M.J., Cantos-Villar E., Faleiro L., Romano A., Medronho B. (2024). Sustainable Extraction of Polyphenols from Vine Shoots Using Deep Eutectic Solvents: Influence of the Solvent, *Vitis* sp., and Extraction Technique. Talanta.

[12] Abbott A.P., Capper G., Davies D.L., Rasheed R.K., Tambyrajah V. (2003). Novel Solvent Properties of Choline Chloride/Urea Mixtures. Chem. Commun..

[13] Kollau L.J.B.M., Vis M., Van Den Bruinhorst A., Esteves A.C.C., Tuinier R. (2018). Quantification of the Liquid Window of Deep Eutectic Solvents. Chem. Commun..

[14] Martins M.A.R., Pinho S.P., Coutinho J.A.P. (2019). Insights into the Nature of Eutectic and Deep Eutectic Mixtures. J. Solut. Chem..

[15] Hansen B.B., Spittle S., Chen B., Poe D., Zhang Y., Klein J.M., Horton A., Adhikari L., Zelovich T., Doherty B.W. (2021). Deep Eutectic Solvents: A Review of Fundamentals and Applications. Chem. Rev..

[16] Dai Y., van Spronsen J., Witkamp G.J., Verpoorte R., Choi Y.H. (2013). Natural Deep Eutectic Solvents as New Potential Media for Green Technology. Anal. Chim. Acta.

[17] Choi Y.H., van Spronsen J., Dai Y., Verberne M., Hollmann F., Arends I.W.C.E., Witkamp G.J., Verpoorte R. (2011). Are Natural Deep Eutectic Solvents the Missing Link in Understanding Cellular Metabolism and Physiology?. Plant Physiol..

[18] Dai Y., Witkamp G.J., Verpoorte R., Choi Y.H. (2015). Tailoring Properties of Natural Deep Eutectic Solvents with Water to Facilitate Their Applications. Food Chem..

[19] Mišan A., Nađpal J., Stupar A., Pojić M., Mandić A., Verpoorte R., Choi Y.H., Mi A., Nad-Pal J., Poji M. (2019). The Perspectives of Natural Deep Eutectic Solvents in Agri-Food Sector. Crit. Rev. Food Sci. Nutr..

[20] Gomez F.J.V., Espino M., Fernández M.A., Silva M.F. (2018). A Greener Approach to Prepare Natural Deep Eutectic Solvents. ChemistrySelect.

[21] Cannavacciuolo C., Pagliari S., Frigerio J., Giustra C.M., Labra M., Campone L. (2023). Natural Deep Eutectic Solvents (NADESs) Combined with Sustainable Extraction Techniques: A Review of the Green Chemistry Approach in Food Analysis. Foods.

[22] Hikmawanti N.P.E., Ramadon D., Jantan I., Mun’im A. (2021). Natural Deep Eutectic Solvents (NADES): Phytochemical Extraction Performance Enhancer for Pharmaceutical and Nutraceutical Product Development. Plants.

[23] Xu P., Zheng G.-W., Zong M.-H., Li N., Lou W.-Y. (2017). Recent Progress on Deep Eutectic Solvents in Biocatalysis. Bioresour. Bioprocess..

[24] Kovács A., Neyts E.C., Cornet I., Wijnants M., Billen P. (2020). Modeling the Physicochemical Properties of Natural Deep Eutectic Solvents. ChemSusChem.

[25] Klamt A. (1995). Conductor-like Screening Model for Real Solvents: A New Approach to the Quantitative Calculation of Solvation Phenomena. J. Phys. Chem..

[26] Klamt A., Jonas V., Bürger T., Lohrenz J.C.W. (1998). Refinement and Parametrization of COSMO-RS. J. Phys. Chem. A.

[27] Eckert F., Klamt A. (2002). Fast Solvent Screening via Quantum Chemistry: COSMO-RS Approach. AIChE J..

[28] Panić M., Gunjević V., Radošević K., Bubalo M.C., Ganić K.K., Redovniković I.R. (2021). COSMOtherm as an Effective Tool for Selection of Deep Eutectic Solvents Based Ready-to-use Extracts from Graševina Grape Pomace. Molecules.

[29] Wojeicchowski J.P., Ferreira A.M., Abranches D.O., Mafra M.R., Coutinho J.A.P. (2020). Using COSMO-RS in the Design of Deep Eutectic Solvents for the Extraction of Antioxidants from Rosemary. ACS Sustain. Chem. Eng..

[30] Zurob E., Cabezas R., Villarroel E., Rosas N., Merlet G., Quijada-Maldonado E., Romero J., Plaza A. (2020). Design of Natural Deep Eutectic Solvents for the Ultrasound-Assisted Extraction of Hydroxytyrosol from Olive Leaves Supported by COSMO-RS. Sep. Purif. Technol..

[31] Zga N., Papastamoulis Y., Toribio A., Richard T., Delaunay J.C., Jeandet P., Renault J.H., Monti J.P., Mérillon J.M., Waffo-Téguo P. (2009). Preparative Purification of Antiamyloidogenic Stilbenoids from *Vitis vinifera* (Chardonnay) Stems by Centrifugal Partition Chromatography. J. Chromatogr. B.

[32] Delaunay J.-C., Castagnino C., Chèze C., Vercauteren J. (2002). Preparative Isolation of Polyphenolic Compounds from *Vitis vinifera* by Centrifugal Partition Chromatography. J. Chromatogr. A.

[33] Sutherland I.A. (2007). Recent Progress on the Industrial Scale-up of Counter-Current Chromatography. J. Chromatogr. A.

[34] Ito Y. (2005). Golden Rules and Pitfalls in Selecting Optimum Conditions for High-Speed Counter-Current Chromatography. J. Chromatogr. A.

[35] Costa F.d.N., Vieira M.N., Garrard I., Hewitson P., Jerz G., Leitão G.G., Ignatova S. (2016). *Schinus terebinthifolius* Countercurrent Chromatography (Part II): Intra-Apparatus Scale-up and Inter-Apparatus Method Transfer. J. Chromatogr. A.

[36] Vieira M.N., Winterhalter P., Jerz G. (2016). Flavonoids from the Flowers of *Impatiens glandulifera* Royle Isolated by High Performance Countercurrent Chromatography. Phytochem. Anal..

[37] DeAmicis C., Edwards N.A., Giles M.B., Harris G.H., Hewitson P., Janaway L., Ignatova S. (2011). Comparison of Preparative Reversed Phase Liquid Chromatography and Countercurrent Chromatography for the Kilogram Scale Purification of Crude Spinetoram Insecticide. J. Chromatogr. A.

[38] Hopmann E., Arlt W., Minceva M. (2011). Solvent System Selection in Counter-Current Chromatography Using Conductor-like Screening Model for Real Solvents. J. Chromatogr. A.

[39] Hopmann E., Frey A., Minceva M. (2012). A Priori Selection of the Mobile and Stationary Phase in Centrifugal Partition Chromatography and Counter-Current Chromatography. J. Chromatogr. A.

[40] Frey A., Hopmann E., Minceva M. (2014). Selection of Biphasic Liquid Systems in Liquid-Liquid Chromatography Using Predictive Thermodynamic Models. Chem. Eng. Technol..

[41] Bezold F., Weinberger M.E., Minceva M. (2017). Computational Solvent System Screening for the Separation of Tocopherols with Centrifugal Partition Chromatography Using Deep Eutectic Solvent-Based Biphasic Systems. J. Chromatogr. A.

[42] Kiene M., Blum S., Jerz G., Winterhalter P. (2023). A Comparison between High-Performance Countercurrent Chromatography and Fast-Centrifugal Partition Chromatography for a One-Step Isolation of Flavonoids from Peanut Hulls Supported by a Conductor like Screening Model for Real Solvents. Molecules.

[43] ChemSpider Royal Society of Chemistry. http://www.chemspider.com.

[44] BIOVIA COSMOtherm, Release 2022; Dassault Systèmes. http://www.3ds.com.

[45] Shen Z., Van Lehn R.C. (2020). Solvent Selection for the Separation of Lignin-Derived Monomers Using the Conductor-like Screening Model for Real Solvents. Ind. Eng. Chem. Res..

[46] Brace E.C., Engelberth A.S. (2017). Enhancing Silymarin Fractionation Using the Conductor-like Screening Model for Real Solvents. J. Chromatogr. A.

[47] Luca S.V., Roehrer S., Kleigrewe K., Minceva M. (2020). Approach for Simultaneous Cannabidiol Isolation and Pesticide Removal from Hemp Extracts with Liquid-Liquid Chromatography. Ind. Crop. Prod..

[48] Diedenhofen M., Klamt A. (2010). COSMO-RS as a Tool for Property Prediction of IL Mixtures—A Review. Fluid Phase Equilib..

[49] Wilkens A. (2011). Isolierung und Strukturaufklärung von Oligomeren Stilbenen aus Weinrebenextrakten.

[50] Stahl E., Kaltenbach U. (1961). Dünnschicht-Chromatographie: VI. Mitteilung. Spurenanalyse von Zuckergemischen Auf Kieselgur G-Schichten. J. Chromatogr. A.

[51] Brent Friesen J., Pauli G.F. (2005). G.U.E.S.S.—A Generally Useful Estimate of Solvent Systems for CCC. J. Liq. Chromatogr. Relat. Technol..

[52] Berthod A., Friesen J.B., Inui T., Pauli G.F. (2007). Elution–Extrusion Countercurrent Chromatography: Theory and Concepts in Metabolic Analysis. Anal. Chem..

[53] Abranches D.O., Silva L.P., Martins M.A.R., Pinho S.P., Coutinho J.A.P. (2020). Understanding the Formation of Deep Eutectic Solvents: Betaine as a Universal Hydrogen Bond Acceptor. ChemSusChem.

[54] Silva L.P., Fernandez L., Conceiçao J.H.F., Martins M.A.R., Sosa A., Ortega J., Pinho S.P., Coutinho J.A.P. (2018). Design and Characterization of Sugar-Based Deep Eutectic Solvents Using Conductor-like Screening Model for Real Solvents. ACS Sustain. Chem. Eng..

[55] Romero-Pérez A.I., Lamuela-Raventós R.M., Andrés-Lacueva C., de la Torre-Boronat M.C. (2001). Method for the Quantitative Extraction of Resveratrol and Piceid Isomers in Grape Berry Skins. Effect of Powdery Mildew on the Stilbene Content. J. Agric. Food Chem..

[56] Rusjan D., Mikulic-Petkovsek M. (2015). Phenolic Responses in 1-Year-Old Canes of *Vitis vinifera* cv. Chardonnay Induced by Grapevine Yellows (Bois Noir). Aust. J. Grape Wine Res..

[57] Dai Y., Rozema E., Verpoorte R., Choi Y.H. (2016). Application of Natural Deep Eutectic Solvents to the Extraction of Anthocyanins from *Catharanthus roseus* with High Extractability and Stability Replacing Conventional Organic Solvents. J. Chromatogr. A.

[58] Vinatoru M. (2001). An Overview of the Ultrasonically Assisted Extraction of Bioactive Principles from Herbs. Ultrason. Sonochem..

[59] Wojeicchowski J.P., Marques C., Igarashi-Mafra L., Coutinho J.A.P., Mafra M.R. (2021). Extraction of Phenolic Compounds from Rosemary Using Choline Chloride–Based Deep Eutectic Solvents. Sep. Purif. Technol..

