# Supplementary material for: In Silico-Assisted Isolation of trans-Resveratrol and trans-ε-Viniferin from Grapevine Canes and Their Sustainable Extraction Using Natural Deep Eutectic Solvents (NADES)
Source: Foods. 2023 Nov 20;12(22):4184. doi: 10.3390/foods12224184 (PMC10670976; doi:10.3390/foods12224184)
Supplement: Supplementary file 1 [file foods-12-04184-s001.zip › foods-2711448-supplementary.docx]

**Supplementary Material**

**In silico-assisted Isolation of *trans*-Resveratrol and *trans*-ε-Viniferin from Grapevine Canes and their Sustainable Extraction using Natural Deep Eutectic Solvents (NADES)**

Mats Kiene ^1^, Malte Zaremba ^1^, Hendrik Fellensiek ^1^, Edwin Januschewski ^1,2^, Andreas Juadjur ^2^, Gerold Jerz ^1^ and Peter Winterhalter ^1,^*

^1^ Institute of Food Chemistry, Technische Universität Braunschweig, Schleinitzstraße 20,
38106 Braunschweig, Germany; m.kiene@tu-braunschweig.de (M.K.); e.januschewski@dil-ev.de (E.J.); g.jerz@tu-braunschweig.de (G.J.)

^2^ German Institute of Food Technologies, Chemical Analytics, Prof.-von-Klitzing-Straße 7,
49610 Quakenbrück, Germany; a.juadjur@dil-ev.de

* Correspondence: p.winterhalter@tu-braunschweig.de

**Section S1: Calculated data by COSMOtherm**

**Figure S1.** Sigma profiles obtained by COSMOthermX of resveratrol (**–**) and ε-viniferin (**–**).

**Table S1.** COSMOthermX (Version 22.0.0) phase equilibrium data for HPCCC solvent system selection at 20 °C with the TZVPD-FINE parametrization.

| **upper phase** | | | | | **lower phase** | | | | |
| --- | --- | --- | --- | --- | --- | --- | --- | --- | --- |
| **HEMWat system** | ***n*-hexane** | **ethyl acetate** | **methanol** | **water** | | ***n*-hexane** | **ethyl acetate** | **methanol** | **water** |
| 1 | 0.32945405 | 0.44680846 | 0.16395232 | 0.05978516 | | 6.19E-04 | 0.04904734 | 0.27067095 | 0.67966285 |
| 2 | 0.21273348 | 0.48238791 | 0.20430473 | 0.10057388 | | 5.57E-04 | 0.04980333 | 0.26174817 | 0.68789161 |
| 3 | 0.33168729 | 0.48190922 | 0.12651999 | 0.0598835 | | 2.44E-04 | 0.03810865 | 0.20204728 | 0.75960013 |
| 4 | 0.22244545 | 0.52711194 | 0.15441311 | 0.0960295 | | 2.24E-04 | 0.03940768 | 0.19592717 | 0.76444130 |

**Table S2.** Activity coefficients ln *γ* calculated using COSMO-RS (25 °C) of resveratrol in different NADES. The molar ratio for choline chloride and betaine-based systems is 3/1, while that for glucose- and fructose-based systems is 1/3. The water content for all NADES is 0 wt%. Abbreviations: AA, acetic acid; AdA, adipic acid; BA, benzoic acid; B, betaine; Bdiol, 1,4-butanediol; Ch, choline chloride; CA, citric acid; Fru, fructose; Glu, glucose; Gly, glycerol; LA, lactic acid; MA, malic acid; OxA, oxalic acid; Pdiol, 1,2-propanediol; Sor, sorbitol; Suc, sucrose; U, urea.

| **HBDs** | **HBAs** | | | |
| --- | --- | --- | --- | --- |
|  | **Ch** | **B** | **Glu** | **Fru** |
| Suc | –7.28404 | –12.9634 | –2.67041 | –3.21234 |
| MA | –6.42025 | –18.76172 | –0.9885 | –1.70571 |
| Sor | –9.15013 | –18.50369 | –4.08502 | –4.90174 |
| U | –7.76871 | –17.16287 | –1.58102 | –2.50633 |
| CA | –5.31289 | –17.31433 | –0.82378 | –1.56548 |
| AdA | –9.75037 | –18.69583 | –1.59258 | –2.44408 |
| BA | –11.00973 | –20.4778 | –1.56039 | –2.54486 |
| Pdiol | –10.5505 | –19.87727 | –4.86889 | –6.37516 |
| AA | –8.76969 | –19.91218 | –2.05986 | –3.35889 |
| LA | –7.3663 | –19.34018 | –1.94327 | –2.32783 |
| OxA | –6.32495 | –17.46756 | –0.05724 | –1.02256 |
| Bdiol | –8.89575 | –18.94764 | –6.92555 | –9.05403 |
| Gly | –8.13391 | –17.43897 | –3.25435 | –3.39167 |

**Table S3.** Activity coefficients ln *γ* calculated using COSMO-RS (25 °C) of ε-viniferin in different NADES. The molar ratio for choline chloride and betaine-based systems is 3/1, while that for glucose- and fructose-based systems is 1/3. The water content for all NADES is 0 wt%. Abbreviations: AA, acetic acid; AdA, adipic acid; BA, benzoic acid; B, betaine; Bdiol, 1,4-butanediol; Ch, choline chloride; CA, citric acid; Fru, fructose; Glu, glucose; Gly, glycerol; LA, lactic acid; MA, malic acid; OxA, oxalic acid; Pdiol, 1,2-propanediol; Sor, sorbitol; Suc, sucrose; U, urea.

| **HBDs** | **HBAs** | | | |
| --- | --- | --- | --- | --- |
|  | **Ch** | **B** | **Glu** | **Fru** |
| Suc | –10.8241 | –22.09596 | –4.49335 | –5.35344 |
| MA | –8.53072 | –32.08825 | –0.31366 | –0.82378 |
| Sor | –13.65704 | –31.74355 | –6.98985 | –8.31139 |
| U | –10.21304 | –29.24558 | –3.30829 | –4.36388 |
| CA | –6.72329 | –29.60787 | 0.37897 | –0.10171 |
| AdA | –14.49017 | –32.12468 | –2.68894 | –3.99526 |
| BA | –16.98577 | –35.26749 | –2.7715 | –4.16309 |
| Pdiol | –15.58572 | –34.08944 | –8.67963 | –11.19948 |
| AA | –10.68699 | –34.07668 | –3.87587 | –5.87782 |
| LA | –9.96071 | –33.12803 | –3.73336 | –4.24072 |
| OxA | –7.83618 | –29.91451 | –0.54184 | –1.83266 |
| Bdiol | –12.09794 | –32.40208 | –12.03129 | –15.70017 |
| Gly | –11.19993 | –29.80649 | –5.9088 | –6.16332 |

**Section S2: Calculation of countercurrent chromatographic separation parameters, TLC screening of HPCCC fractions, ESI-MS/MS and NMR data of isolated compounds**

The chromatographic elution time was converted over *elution/retention volumes V_R_* into their respective *partition ratio values K_D_* (cf. Equations S1-S8). The *K_D_*-based projection enables a better comparison between different liquid/liquid chromatography based machine designs such as high-performance countercurrent chromatography (HPCCC). The experimental *V_R_*-values of the two stilbenoids resveratrol and *ε*-viniferin from the HPCCC runs were screened by thin-layer chromatography (TLC) and calculated with Equation S1.

***Retention volume V_R_ = elution time [min] x flow rate [mL/min]* (Eq. S1)**

The *S_F_-values* of the used solvent systems were determined by Equations S2a-b using *V_C_* (HPCCC: 125 mL), and *V_M_* resulting in the *S_F_-value* measured at the hydrodynamic equilibrium.

*V_S_* = (*V_C_* – *V_M_*) **(Eq. S2a)**

*S_F_* = *V_S_* / *V_C_* x 100% **(Eq. S2b)**

HPCCC A: *S_F_* = 68%

HPCCC B: *S_F_* = 76%

*V_S_*: retained experimental stationary phase volume

*V_C_*: column volume/capacity (HPCCC: 125 mL)

*V_M_*: volume of mobile phase take up to the coil at equilibrium of HPCCC

*S_F_*: stationary phase retention

*K_D_*: partition ratio

The determined *S_F_-value* in the experiments is corrected by the *extra column volume V_ext_* (HPCCC: 7 mL) [1] of the connecting periphery tubing in the HPCCC set-up, using Equations S3-S5

**Corrected *V_M_* = *V_M_* - *V_ext_* (Eq. S3)**

HPCCC A: *corr. V_M_* = 40 mL - 7 mL = 33 mL

HPCCC B: *corr. V_M_* = 30 mL - 7 mL = 23 mL

**Corrected *V_S_* = *V_C_* – corrected *V_M_* (Eq. S4)**

HPCCC A: *corr. V_S_* = 125 mL - 33 mL = 92 mL

HPCCC B: *corr. V_S_* = 125 mL - 23 mL = 102 mL

**Corrected *S_F_* = corrected *V_S_* / *V_C_* (Eq. S5)**

HPCCC A: *corr. S_F_* = 92 mL / 125 mL x 100% = 74%

HPCCC B: *corr. S_F_* = 102 mL / 125 mL x 100% = 82%

It should be noted that a high *S_F_-value* directly correlate to a higher resolution and efficiency of the HPCCC separation.

The compound and solvent system specific partition ratio *K_D_*-values in the HPCCC run were calculated by the Equation S6.

During *elution-mode*: [2]

***K_D_ = (V_R_ – corrected V_M_) / corrected V_S_* (Eq. S6)**

The *separation factor α* and *resolution factor* *R_S_* depend on the distances between peaks and the peak widths that will be compared. The calculation of both factors depend on the determined *K_D_*-values. *α* describes the ability of HPCCC system to distinguish between sample components (Eq. S7).

***α = K_D2_ / K_D1_ (with K_D2_ > K_D1_)*** **(Eq. S7)**

The calculation of the *resolution factor* *R_S_* described how well two peaks are separated at the baseline from each other (Eq. S8).

***R_S_ = 2 (K_D2_ - K_D1_) / (W_2_ + W_1_)*** **(Eq. S8)**

W_n_: peak width at baseline

**Table S4.** Compound specific *K_D_*-value from *heart-cut fraction* of HPCCC separation A.

| **HPCCC A** | **Corrected *S_F_*** | **74%** |  |
| --- | --- | --- | --- |
| **Compounds** | **Exp. Peak Range**  **Fractions**  **Retention Vol. [mL]**  **Peak Width [mL]** | ***K_D_* Range**  ***∆K_D_* Width W** | **HPCCC**  **Mean Value**  *x̅* ***K_D_*** |
| *heart-cut fraction* | F52-F65  208-260  52 | 1.90-2.46  0.56 | 2.18 |

**Table S5.** Target compound specific *K_D_*-values from HPCCC separation B.

| **HPCCC B** | **Corrected *S_F_*** | **82%** |  |
| --- | --- | --- | --- |
| **Target compounds** | **Exp. Peak Range**  **Fractions**  **Retention Vol. [mL]**  **Peak Width [mL]** | ***K_D_* Range**  ***∆K_D_* Width W** | **HPCCC**  **Mean Value**  *x̅* ***K_D_*** |
| ε-viniferin | F18-F27  54-81  27 | 0.30-0.56  0.26 | 0.44 |
| resveratrol | F33-F40  99-120  21 | 0.75-0.95  0.20 | 0.85 |

**Figure S2.** TLC analysis of the HPCCC fractions. Normal phase silica gel TLC plates (Merck GmbH, Darmstadt, Germany) were developed with chloroform/ ethyl acetate/ methanol/ water (25/55/5/1; *v/v/v/v*). Visualization was done using spray reagent anisaldehyde-sulfuric acid-glacial acid (universal reagent Egon Stahl [3]) and thermo-development (105 °C).

**ESI-MS/MS and NMR data of isolated compounds**

**Table S6.** ESI-MS/MS data of isolated compounds.

| ***comp.*** | ***MW [g/mol]*** | ***ESI polarity*** | ***pseudo molecular ion*** | ***parent ion m/z*** | ***m/z from MS²*** |
| --- | --- | --- | --- | --- | --- |
| resveratrol | 228 | neg. | [M-H]^-^ | 227 | 185, 175, 159 |
| ε-viniferin | 454 | neg. | [M-H]^-^ | 453 | 435, 411, 359, 347, 289, 253, 225 |

^a^ Base peaks are underlined, MS² relative intensities in brackets

**Figure S3.** Structure of *trans*-resveratrol.

**Table S7.** NMR data of *trans*-resveratrol.^a^

| **Position**^b^ | **^13^C: *δ_C_* [ppm]** | **^1^H: *δ_H_* [ppm]; mult., *J* [Hz]** |
| --- | --- | --- |
| **1** | 140.8 | q |
| **2** | 105.6 | 6.55; d, *J* = 2.2 |
| **3** | 159.5 | q |
| **4** | 102.6 | 6.27; t, *J* = 2.2 |
| **5** | 159.5 | q |
| **6** | 105.6 | 6.55; d, *J* = 2.2 |
| **7** | 126.7 | 6.89; d, *J* = 16.3 |
| **8** | 129.0 | 7.02; d, *J* = 16.3 |
| **1’** | 129.9 | q |
| **2’** | 128.8 | 7.43; m |
| **3’** | 116.3 | 6.84; m |
| **4’** | 158.1 | q |
| **5’** | 116.3 | 6.84; m |
| **6’** | 128.8 | 7.43; m |

^a^ Solvent: acetone-*d6*, tetramethylsilane *δ* 0.00 ppm for ^1^H, *δ* 29.8 ppm for ^13^C; ^1^H observed frequency 300.1 MHz, ^13^C observed frequency 75.5 MHz.

^b^ For numbering of the carbon atoms, see the formula, assignment of C-H via HSQC data.

**Figure S4.** ^1^H-NMR spectrum of *trans*-resveratrol.

**Figure S5.** ^13^C-NMR spectrum of *trans*-resveratrol.

**Figure S6.** HSQC spectrum of *trans*-resveratrol.

**Figure S7. HMBC** spectrum of *trans*-resveratrol.

**Figure S8.** Structure of *trans*-ε-viniferin.

**Table S8.** NMR data of *trans*-ε-viniferin.^a^

| **Position**^b^ | **^13^C: *δ_C_* [ppm]** | **^1^H: *δ_H_* [ppm]; mult., *J* [Hz]** |
| --- | --- | --- |
| **1a** | 133.8 | q |
| **2a** | 127.9 | 7.21; m, *J* = 8.6 |
| **3a** | 116.0 | 6.83; m, *J* = 8.6 |
| **4a** | 158.1 | q |
| **5a** | 116.0 | 6.83; m, *J* = 8.6 |
| **6a** | 127.9 | 7.21; m, *J* = 8.6 |
| **7a** | 93.8 | 5.43; d, *J* = 5.5 |
| **8a** | 57.0 | 4.49; d, *J* = 5.5 |
| **9a** | 147.4 | q |
| **10a** | 106.8 | 6.25; s |
| **11a** | 159.7 | q |
| **12a** | 101.9 | 6.25; s |
| **13a** | 159.7 | q |
| **14a** | 106.8 | 6.25; s |
| **1b** | 129.8 | q |
| **2b** | 128.7 | 7.18; m, *J* = 8.6 |
| **3b** | 116.2 | 6.74; m, *J* = 8.6 |
| **4b** | 158.1 | q |
| **5b** | 116.2 | 6.74; m, *J* = 8.6 |
| **6b** | 128.7 | 7.18; m, *J* = 8.6 |
| **7b** | 130.0 | 6.92; d, *J* = 16.4 |
| **8b** | 123.4 | 6.72; d, *J* = 16.3 |
| **9b** | 136.3 | q |
| **10b** | 119.8 | q |
| **11b** | 162.4 | q |
| **12b** | 96.6 | 6.33; d, *J* = 2.0 |
| **13b** | 159.5 | q |
| **14b** | 104.0 | -^c^ |

^a^ Solvent: acetone-*d6*, tetramethylsilane *δ* 0.00 ppm for ^1^H, *δ* 29.8 ppm for ^13^C; ^1^H observed frequency 300.1 MHz, ^13^C observed frequency 75.5 MHz.

^b^ For numbering of the carbon atoms, see the formula, assignment of C-H via HSQC data.

^c^ Signal 14b was superimposed by signals 3b/5b/8b in the ^1^H-NMR spectrum.

**Figure S9.** ^1^H-NMR spectrum of *trans*-*ε*-viniferin.

**Figure S10.** ^13^C-NMR spectrum of *trans*-*ε*-viniferin.

**Figure S11.** HSQC spectrum of *trans*-ε-viniferin.

**Figure S12.** HMBC spectrum of *trans*-ε-viniferin.

**Section S3: Experimental data of NADES extraction**

**Table S9.** Extraction contents of resveratrol and ε-viniferin in different extractants. Abbreviations: B, betaine; Bdiol, 1,4-butanediol; Ch, choline chloride; Glu, glucose; Pdiol, 1,2-propanediol; Sor, sorbitol; U, urea.

| **Extractant** | **resveratrol** | ***ε*-viniferin** |
| --- | --- | --- |
| EtOH/H_2_O four times | 4.40 ± 0.04 | 3.11 ± 0.03 |
| EtOH/H_2_O one time | 3.99 ± 0.12 | 2.87 ± 0.06 |
| Ch/Pdiol 1/5, 0 wt% H_2_O | 3.96 ± 0.16 | 2.88 ± 0.21 |
| B/Bdiol 1/1, 30 wt% H_2_O | 3.96 ± 0.08 | 2.76 ± 0.05 |
| B/Sor 1/1, 30 wt% H_2_O | 1.90 ± 0.06 | 1.61 ± 0.09 |
| Glu/U 1/3, 30 wt% H_2_O | 0.87 ± 0.09 | 0.74 ± 0.10 |
| Glu/U 1/1, 60 wt% H_2_O | 0.55 ± 0.10 | 0.44 ± 0.07 |

Results expressed as mean ± standard deviation (milligrams per gram of dry weight).

**References**

1. Costa, F. das N.; Vieira, M.N.; Garrard, I.; Hewitson, P.; Jerz, G.; Leitão, G.G.; Ignatova, S. Schinus Terebinthifolius Countercurrent Chromatography (Part II): Intra-Apparatus Scale-up and Inter-Apparatus Method Transfer. *J. Chromatogr. A* **2016**, *1466*, 76–83.

2. Berthod, A.; Friesen, J.B.; Inui, T.; Pauli, G.F. Elution−Extrusion Countercurrent Chromatography: Theory and Concepts in Metabolic Analysis. *Anal. Chem.* **2007**, *79* (9), 3371–3382.

3. Stahl, E.; Kaltenbach, U. Dünnschicht-Chromatographie: VI. Mitteilung. Spurenanalyse von Zuckergemischen Auf Kieselgur G-Schichten. *J. Chromatogr. A* **1961**, *5* (1961), 351–355.
